# Nationwide registry of glecaprevir plus pibrentasvir in the treatment of HCV in Taiwan

**DOI:** 10.1038/s41598-021-03006-3

**Published:** 2021-12-06

**Authors:** Chung-Feng Huang, Hsing-Tao Kuo, Te-Sheng Chang, Ching-Chu Lo, Chao-Hung Hung, Chien-Wei Huang, Lee-Won Chong, Pin-Nan Cheng, Ming-Lun Yeh, Cheng-Yuan Peng, Chien-Yu Cheng, Jee-Fu Huang, Ming-Jong Bair, Chih-Lang Lin, Chi-Chieh Yang, Szu-Jen Wang, Tsai-Yuan Hsieh, Tzong-Hsi Lee, Pei-Lun Lee, Wen-Chih Wu, Chih-Lin Lin, Wei-Wen Su, Sheng-Shun Yang, Chia-Chi Wang, Jui-Ting Hu, Lein-Ray Mo, Chun-Ting Chen, Yi-Hsiang Huang, Chun-Chao Chang, Chia-Sheng Huang, Guei-Ying Chen, Chien-Neng Kao, Chi-Ming Tai, Chun-Jen Liu, Mei-Hsuan Lee, Pei-Chien Tsai, Chia-Yen Dai, Jia-Horng Kao, Han-Chieh Lin, Wang-Long Chuang, Chi-Yi Chen, Kuo-Chih Tseng, Ming-Lung Yu

**Affiliations:** 1 Hepatobiliary Division, Department of Internal Medicine and Hepatitis Center, Kaohsiung Medical University Hospital, Kaohsiung Medical University, Kaohsiung, Taiwan; 2 Division of Gastroenterology and Hepatology, Department of Internal Medicine, Chi Mei Medical Center, Yongkang District, Tainan, Taiwan; 3 Division of Hepatogastroenterology, Department of Internal Medicine, ChiaYi Chang Gung Memorial Hospital and College of Medicine, Chang Gung University, Taoyuan, Taiwan; 4 Division of Gastroenterology, Department of Internal Medicine, St. Martin De Porres Hospital, Chiayi, Taiwan; 5 Division of Gastroenterology, Kaohsiung Armed Forces General Hospital, Kaohsiung, Taiwan; 6 Division of Hepatology and Gastroenterology, Department of Internal Medicine, Shin Kong Wu Ho-Su Memorial Hospital, Taipei, Taiwan; 7 School of Medicine, Fu-Jen Catholic University, New Taipei City, Taiwan; 8 Division of Gastroenterology and Hepatology, Department of Internal Medicine, National Cheng Kung University Hospital, College of Medicine, National Cheng Kung University, Tainan, Taiwan; 9 Department of Internal Medicine, Kaohsiung Municipal Siaogang Hospital, Kaohsiung Medical University Hospital, Kaohsiung Medical University, Kaohsiung, Taiwan; 10 Center for Digestive Medicine, Department of Internal Medicine, China Medical University Hospital, Taichung, Taiwan; 11 School of Medicine, China Medical University, Taichung, Taiwan; 12 Division of Infectious Diseases, Department of Internal Medicine, Taoyuan General Hospital, Ministry of Health and Welfare, Taoyuan, Taiwan; 13 Deppartment of Internal Medicine, Kaohsiung Municipal Ta-Tung Hospital, Kaohsiung Medical University Hospital, Kaohsiung Medical University, Kaohsiung, Taiwan; 14 Division of Gastroenterology, Department of Internal Medicine, Taitung Mackay Memorial Hospital, Taitung, Taiwan; 15 Mackay Medical College, New Taipei City, Taiwan; 16 Liver Research Unit, Department of Hepato-Gastroenterology and Community Medicine Research Center, Chang Gung Memorial Hospital at Keelung, College of Medicine, Chang Gung University, Keelung, Taiwan; 17 Department of Gastroenterology, Division of Internal Medicine, Show Chwan Memorial Hospital, Changhua, Taiwan; 18 Division of Gastroenterology, Department of Internal Medicine, Yuan’s General Hospital, Kaohsiung, Taiwan; 19 Division of Gastroenterology, Department of Internal Medicine, Tri-Service General Hospital, National Defense Medical Center, Taipei, Taiwan; 20 Division of Gastroenterology and Hepatology, Far Eastern Memorial Hospital, New Taipei City, Taiwan; 21 Division of Gastroenterology and Hepatology, Department of Internal Medicine, Chi Mei Medical Center, Liouying, Tainan, Taiwan; 22 Wen-Chih Wu Clinic, Fengshan, Kaohsiung, Taiwan; 23 Department of Gastroenterology, Renai Branch, Taipei City Hospital, Taipei, Taiwan; 24 Department of Gastroenterology and Hepatology, Changhua Christian Hospital, Changhua, Taiwan; 25 Division of Gastroenterology and Hepatology, Department of Internal Medicine, Taichung Veterans General Hospital, Taichung, Taiwan; 26 Taipei Tzu Chi Hospital, Buddhist Tzu Chi Medical Foundation and School of Medicine, Tzu Chi University, Taipei, Taiwan; 27 Liver Center, Cathay General Hospital, Taipei, Taiwan; 28 Division of Gastroenterology, Tainan Municipal Hospital (Managed By Show Chwan Medical Care Corporation), Tainan, Taiwan; 29 Division of Gastroenterology, Department of Internal Medicine Tri-Service General Hospital Penghu Branch, National Defense Medical Center, Taipei, Taiwan; 30 Division of Gastroenterology and Hepatology, Department of Medicine, Taipei Veterans General Hospital, Taipei, Taiwan; 31 Institute of Clinical Medicine, School of Medicine, National Yang-Ming Chiao Tung University, Taipei, Taiwan; 32 Division of Gastroenterology and Hepatology, Department of Internal Medicine, Taipei Medical University Hospital, Taipei, Taiwan; 33 Division of Gastroenterology and Hepatology, Department of Internal Medicine, School of Medicine, College of Medicine, Taipei Medical University, Taipei, Taiwan; 34 Yang Ming Hospital, Chiayi, Taiwan; 35 Penghu Hospital, Ministry of Health and Welfare, Penghu, Taiwan; 36 National Taiwan University Hospital Hsin-Chu Branch, Hsinchu, Taiwan; 37 Department of Internal Medicine, E-Da Hospital, Kaohsiung, Taiwan; 38 School of Medicine, College of Medicine, I-Shou University, Kaohsiung, Taiwan; 39 Hepatitis Research Center and Department of Internal Medicine, National Taiwan University Hospital, Taipei, Taiwan; 40 Institute of Clinical Medicine, National Yang-Ming Chiao Tung University, Taipei, Taiwan; 41 Division of Gastroenterology and Hepatology, Department of Medicine, Ditmanson Medical Foundation Chiayi Christian Hospital, Chiayi, Taiwan; 42 Department of Internal Medicine, Dalin Tzu Chi Hospital, Buddhist Tzu Chi Medical Foundation, Chiayi, Taiwan; 43 School of Medicine, Tzuchi University, Hualien, Taiwan

**Keywords:** Microbiology, Diseases, Gastroenterology

## Abstract

The study evaluated the real-world treatment outcomes of Glecaprevir/pibrentasvir (GLE/PIB) including effectiveness, safety and healthcare resource utilization based on a nation-wide registry in Taiwan. The Taiwan HCV Registry **(**TACR) is a nation-wide platform organized and supervised by the Taiwan Association for the Study of the Liver. Data were analyzed for patients treated with GLE/PIB, including 3144 patients who had treatment outcome available. The primary endpoint was sustained virological response (SVR12, undetectable HCV RNA throughout 12 weeks of end-of-treatment). The overall SVR12 rate was 98.9% (3110/3144), with 98.8%, 99.4% and 100% in patients receiving 8 weeks, 12 weeks, and 16 weeks of GLE/PIB respectively. The SVR12 rate in the treatment-naïve cirrhotic patients receiving 8 weeks of GLE/PIB was 98.2% (108/110). The most common AEs were fatigue (7.5%), pruritus (6.7%) and dizziness (1.5%). The mean number of outpatient visits during the GLE/PIB was 5.94 visits for patients treated with 8 weeks, significantly different from the patients treated with 12 weeks of GLE/PIB (6.90 visits). The results support the effectiveness and safety of GLE/PIB treatment in real-world clinical practice, and provide further evidence that the shorter, 8-week GLE/PIB regimen is effective and cost-saving.

## Introduction

Hepatitis C virus (HCV) infection is recognized as a global health threat, affecting approximately 71 million patients worldwide^1^. In Taiwan, HCV is endemic with an estimated prevalence ranged from 3.28% among the general population to more than 10% in hyperendemic areas^2,3^. One of the five core interventions identified by the World Health Organization (WHO) toward eliminating viral hepatitis is to enhance and expand the response of the oral, well-tolerated direct-acting antivirals (DAAs) for people with chronic hepatitis C virus (CHC) infection, which can achieve cure rates of over 90% and thereafter avoid further transmission and reduce HCV-related complications, including decompnesated cirrhosis, liver transplantations and death^1,4^.

Among the all-oral DAAs recommended by the regulations of the Health and Welfare Department of Taiwan^5^ and regional guidelines^6,7^, the regimen of glecaprevir/pibrentasvir (GlE/PIB) provides the opportunity for shortening CHC therapy to 8 weeks in the majority of patients^8^. GLE/PIB is a once daily, ribavirin-free, fixed-dose combination of two pangeotypic DAAs: glecaprevir (a HCV NS3/4A protease inhibitor) and pibrentasvir (a NS5A inhibitor), indicated for CHC patients for a duration of 8, 12 or 16 weeks^9^. The efficacy and safety data of GLE/PIB for the indicated CHC patients have been demonstrated in registrational phase II, phase III clinical trials and in a number of European reports^8–11^; however, there are only limited data evaluating the efficacy of GLE/PIB in real-world, non-clinical trial settings. Since the Taiwan National Health Insurance (NHI) program started to reimburse GLE/PIB in August 2018, several real-world data regarding the effectiveness and safety of DAAs including GLE/PIB have been reported from Taiwan^12–15^. As nation-wide data of GLE/PIB, especially from special populations, are scarce, the aim of the present study was to evaluate the real-world efficacy and safety of GLE/PIB in adult patient with CHC infection enrolled in the Taiwan Association for the Study of the Liver HCV Registry (TACR).

## Methods

### Patients and study design

The TASL HCV Registry **(**TACR) is an ongoing, non-interventional, prospective, observational nationwide cohort study organized and funded by the Taiwan Association for the Study of the Liver (TASL), which set up and manages the database and biobank of HCV patients who receive DAA therapy in Taiwan, as previously described^15^. The study protocol was approved by the Institutional Review Board of Kaohsiung Medical University Hospital, which conformed to the guidelines of the International Conference on Harmonization for Good Clinical Practice. All patients had to provide written informed consent before being enrolled in the registry. Inclusion criteria for the registry were: 20 years of age or older; chronic HCV infection with detectable HCV ribonucleic acid (RNA) and prescribed with DAA-containing regimens. Individual patient records were reviewed, and data were extracted and validated using a standardized case report form and a unified coding dictionary for the pre-defined patient (e.g., demographics, previous HCV treatment, comorbidities, and cirrhotic status) and virological characteristics (e.g., HCV genotypes, viral loads and treatment outcomes) before and after antiviral treatment. In this registry, the choice of DAA was at the discretion of the treating physician taking into account the recommendations^6,7^ and the regulations of the Health and Welfare Department of Taiwan^5^. Briefly, apart from the indication of 16-week GlE/PIB for HCV genotype 3 interferon-experienced patients, 8-week GlE/PIB was approved for treatment naïve and interferon-experienced non-cirrhotic patients, whereas 12-week GlE/PIB was approved for treatment naïve and interferon-experienced compensated-cirrhotic patients between February 2018 and March 2020. Since April 2020, 8-week GlE/PIB was further approved for treatment naïve patients with compensated liver cirrhosis**.** The current analysis included patients with CHC infection who received 1 or more dose of GLE/PIB according to the label at the time of registration and had treatment outcome available as of 31 October 2020.

### Assessments and endpoints

Demographics and clinical characteristics were assessed at baseline, including age, gender, HCV genotype, viral load, liver cirrhosis status, history of previous HCV treatment, comorbidities and history of drug abuse. Liver cirrhosis was defined by any of the following: liver histology, transient elastography (FibroScan®; Echosens, Paris, France, > 12 kPa), acoustic radiation force impulse (> 1.98 m/s), fibrosis-4 index (> 6.5) or the presence of clinical, radiological, endoscopic, or laboratory evidence of cirrhosis and/or portal hypertension, as previously described^15^. Hepatocellular carcinoma (HCC) was confirmed by histological or clinical diagnosis, and patients with inactive HCC were defined as those who were subjected to surgical resection, local alcohol injection, radiofrequency ablation or liver transplantation and without imaging evidence of recurrence within 3 months prior to receiving DAA treatment^12^. Patients with chronic kidney disease (CKD) included dialytic patients and patients with a decreased estimated glomerular filtration rate (eGFR, < 60 ml/min/1.73 m^2^) or kidney fucntion damage (e.g., presence of proteinura) for more than 3 months^16^.

Efficacy outcome was the overall rate of sustained virological response (SVR12, defined as undetectable HCV RNA level < lower limit of quantification at off-therapy week 12). SVR12 rates in the following subgroups of interest were evaluated: HCV genotypes (GTs), liver cirrhosis status (no cirrhosis or compensated cirrhosis), previous HCV treatment history (treatment naïve or treatment experienced), comorbidities including hepatitis B virus (HBV)-coinfection, human immunosuppressant virus (HIV)-coinfection, HCC, or CKD, GLE/PIB treatment duration (8, 12, or 16 weeks), adherence (defined as the percentage of actual dosage being taken divided by the anticipated DAA dosage throughout the treatment course in each subject) and history of drug abuse (patient who inject drugs, PWID).

Safety outcomes were the percentages of pateints with adverse events (AEs), serious AEs (SAEs) and common AEs (occurring in ≥ 1% of patients). Healthcare resource utilization (HCRU) was defined as the number of clinic visits from GLE/PIB initiation to the SVR12 survey visit.

### Statistical analyses

Descriptive statistics including mean (± standard deviation, SD) or frequency (percentage) were used to summarize baseline demographics, clinical characteristics and HCRU for each treatment duration. For efficacy outcomes, the overall and stratified viral response rates (SVR12) were shown in numbers and percentages with 95% confidence interval (CI). Frequencies were compared between groups using the χ^2^ test with the Yates correction or Fisher’s exact test. Group means were compared using analysis of variance and Student’s *t*-test or the nonparametric Mann–Whitney U test when appropriate. Stepwise logistic regression analysis was performed to determine factors associated with treatment failure by analyzing the covariates with a *P* value < 0.1 in the univariate analysis. The overall safety profiles were shown in numbers and percentages as appropriate. The statistical analyses were performed using the SPSS 12.0 statistical package (SPSS, Chicago, IL, USA). All statistical analyses were based on two-sided hypothesis tests with a significance level of *P* < 0.05.

## Results

### Patient characteristics

**Figure 1:**
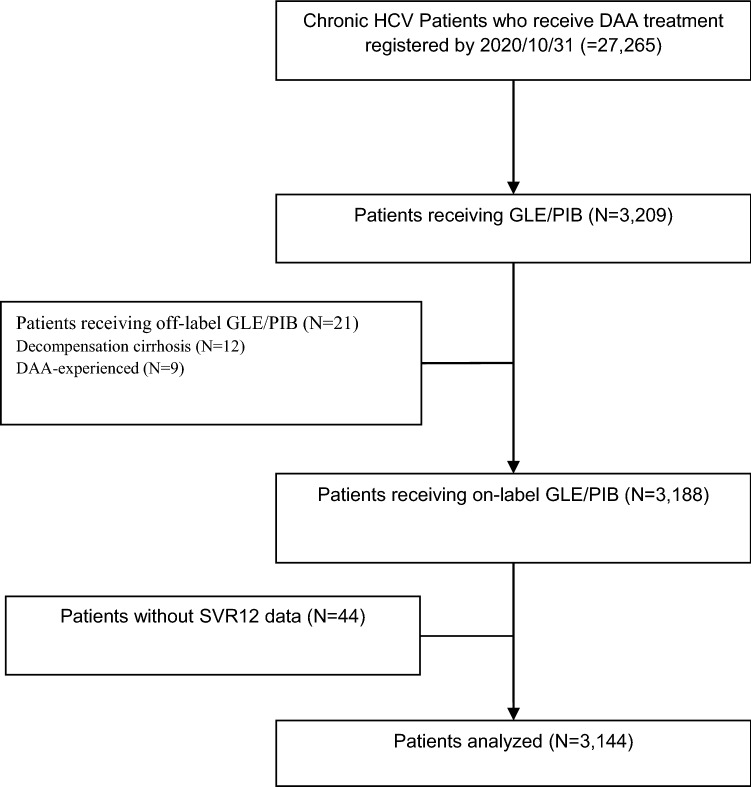
Study Flow. Of the 44 patients without SVR data available, 17 patients terminated treatment earlier, 4 patients passed away and 23 patients lost to follow.

Patient data of a total of 27,265 CHC patients were collected from 48 sites and registered in TACR platform as of 31 October 2020. Among the 3209 patients treated with GLE/PIB, 21 patients were excluded because of documented decompensated cirrhosis at baseline (12 patients) or previous exposure to NS3/4A protease inhibitor and/or NS5A inhibitor-containing DAAs (9 patients), in accordance with the Taiwan Food and Drug Administration (TFDA)-approved label. Of the remaining 3188 patients, 3144 patients with the treatment outcome at post-treatment week 12 available were included in the present analysis (Fig. 1). The mean age was 58.9 years, and females accounted for 49.3% of the population. The dominant viral genotype was HCV genotype 2 (GT2, 56.8%), followed by GT1 (27.5%), GT6 (8.5%) and GT3 (4.6%). Six hundred and eight patients (19.3%) had baseline HCV RNA > 6,000,000 IU/ml. The majority of patients were treatment naïve (92.0%) and had no liver cirrhosis (85.1%). One hundred and seventeen (3.7%) had history of HCC (active, 2.9%; inactive, 0.9%) before DAA treatment; 248 (7.9%) and 154 (4.9%) patients were dually infected with hepatitis B virus (HBV) and human immunodeficiency virus (HIV), respectively; 830 (26.4%) patients had chronic kidney disease (CKD). For high-risk behaviors, 41 patients (1.3%) were documented with a history of intravenous drug abuse. The majority of patients (2601, 82.7%) received GLE/PIB for 8 weeks. Compared to patients receiving 8-week GLE/PIB, those with 12-week regimen were older, had a lower proportion of HCV GT1 and HIV coinfection, and had a higher proportion of CKD, interferon-experienced history, cirrhosis and pre-existing HCC (Table 1).

**Table 1:** Baseline characteristics.

Characteristics	All patients (n = 3144)	8-week (n = 2601)	12-week (n = 522)	16-week (n = 21)	*P* value (8 week vs.12 week)
Age (years), mean ± SD	58.9 ± 13.1	58.1 ± 13.2	63.4 ± 12.1	54.7 ± 10.8	< 0.01
Age > 65 y, n (%)	1141 (36.3)	876 (33.7)	260 (49.8)	5 (22.7)	< 0.01
Male, n (%)	1595 (50.7)	1315 (50.6)	267 (51.2)	13 (61.9)	0.81
**HCV genotype**					
1, n (%)	864 (27.5)	741 (28.5)	112 (21.5)	11 (50.0)	< 0.01 (GT1 vs. NonGT1)
2, n (%)	1785 (56.8)	1456 (56.0)	329 (63.0)	0 (0.0)	
3, n (%)	146 (4.6)	105 (4.0)	31 (5.9)	10 (47.6)	
4, n (%)	1 (0.03)	0 (0.0)	1 (0.2)	0 (0.0)	
5, n (%)	1 (0.03)	1 (0.04)	0 (0.0)	0 (0.0)	
6, n (%)	266 (8.5)	239 (9.2)	27 (5.2)	0 (0.0)	
Mixed, n (%)	51 (1.6)	35 (1.4)	16 (3.1)	0 (0.0)	
Unclassified, n (%)	30 (1.0)	24 (0.9)	6 (1.2)	0 (0.0)	
HCV RNA, log_10_ IU/mL, mean ± SD	5.9 ± 1.1	5.9 ± 1.1	5.9 ± 1.0	6.2 ± 0.7	0.44
HCV RNA > 6,000,000 IU/ml, n (%)	608 (19.3)	506 (19.5)	97 (18.6)	5 (23.8)	0.65
**Prior antiviral retreatment**					< 0.01
Naïve, n (%)	2891 (92.0)	2428 (93.3)	458 (87.7)	5 (23.8)	
IFN-based Experienced, n (%)	253 (8.0)	173 (6.7)	64 (12.3)	16 (76.2)	
**Liver cirrhosis**					< 0.01
No, n (%)	2675 (85.1)	2478 (95.3)	188 (36.0)	9 (42.9)	
Yes, n (%)	469 (14.9)	123 (4.7)	334 (64.0)	12 (57.1)	
HBV coinfection, n (%)	248 (7.9)	195 (7.5)	52 (9.9)	1 (4.6)	0.06
HIV coinfection, n (%)	154 (4.9)	148 (5.7)	5 (1.0)	1 (4.8)	< 0.01
**History of HCC**					< 0.01(Non-HCC vs. HCC)
No HCC, n (%)	3027 (96.3)	2561 (98.5)	448 (85.8)	18 (85.7)	
Active HCC, n (%)	90 (2.9)	33 (1.3)	54 (10.3)	3 (14.3)	
Inactive HCC, n (%)	27 (0.9)	7 (0.3)	20 (3.8)	0 (0.0)	
**CKD**					< 0.01
No	2314 (73.6)	1987 (76.4)	310 (59.4)	17 (81.0)	
Yes	830 (26.4)	614 (23.6)	212 (40.6)	4 (19.1)	
PWID, n (%)	41 (1.3)	38 (1.5)	3 (0.6)	0 (0.0)	0.10

HBV, hepatitis B virus; HIV, human immunodeficiency virus; HCC, hepatocellular carcinoma; CKD, chronic kidney disease; PWID, patients who inject drugs; GT, genotype.

### Treatment responses

**Figure 2:**
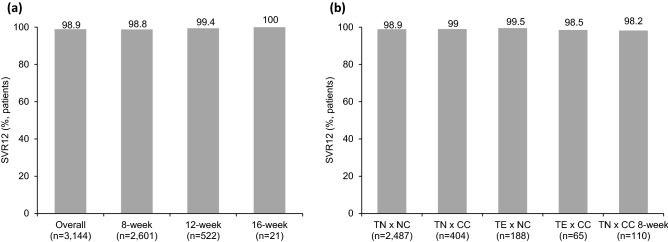
Rate of SVR12 stratified by different treatment durations (**a**), or combinations of previous treatment history and liver cirrhosis status (**b**) (TE, treatment experienced; TN, treatment naïve; CC, compensated cirrhosis; NC, non-cirrhotic).

**Figure 3:**
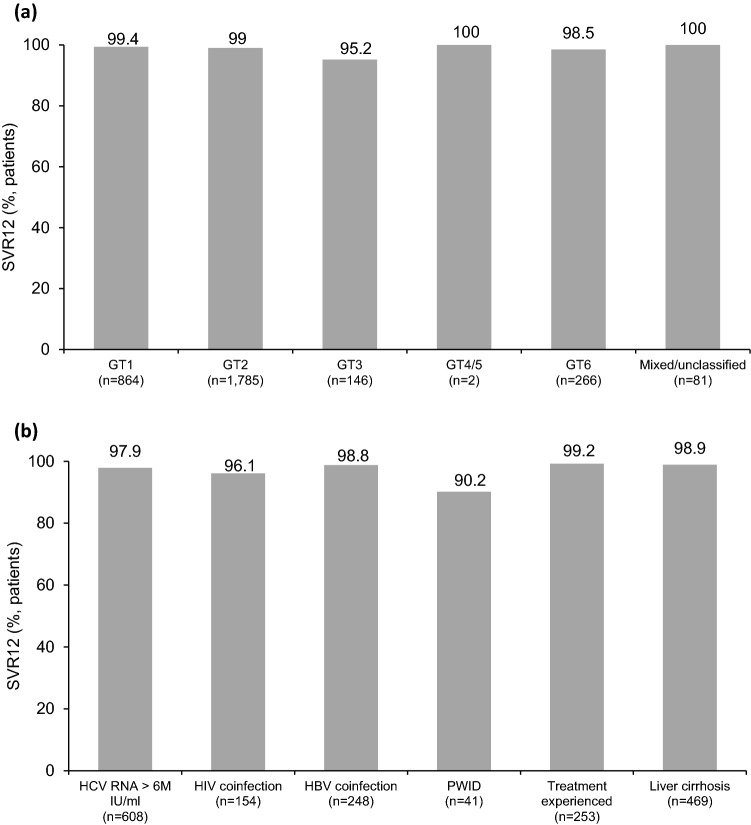
Rate of SVR12 stratified by different genotypes (**a**), or in patient subgroups of interest (**b**) (GT, genotype; PWID, people who inject drugs; HBV, hepatitis B virus; HIV, human immunodeficiency virus).

For the primary efficacy outcome, the overall SVR12 rate was 98.9% (3110/3144). The proportion of SVR12 was 98.8% (2570/2601), 99.4% (519/522), and 100% (21/21) in patients receiving 8, 12 and 16 weeks of treatment, respectively (Fig. 2a). The SVR12 rate was 98.9% (2459/2487), 99.0% (400/404), 99.5% (187/188) and 98.5% (64/65) in treatment-naïve noncirrhotic, treatment-naïve cirrhotic, treatment-experienced noncirrhotic and treatment-experienced cirrhotic patients, respectively (Fig. 2b). When stratified according to HCV genotype, the proportion of SVR12 was 99.4% (2971/2998) in GT1, 99.0% (1767/1785) in GT2, 95.2% (139/146) in GT3, 100% (1/1) in GT4, 100% (1/1) in GT5, 98.5% (262/266) in GT6 and 100% (81/81) in mixed or unclassified genotype (Fig. 3a). Noteworthy, the SVR12 rate was 98.2% (108/110) for the treatment-naïve cirrhotic patients who received GLE/PIB for 8 weeks (Fig. 2b). Four hundred and fifty-seven patients had available data of HCV RNA 4 weeks after the end-of-treatment (SVR4). Of the 455 patients with undetectable HCV RNA at SVR4, 454 achieved SVR12 with the positive predictive value of 99.8%. The other 2 patients with detectable HCV RNA at SVR4 remained viremic at SVR12. All the 12 decompensated patients being excluded were with Child–Pugh B score. Of them, one discontinued therapy at treatment week 2 due to severe constipation, and 2 patients lost follow-up. All the other 9 patients who completed treatment achieved SVR12 and none died. Seven of the 9 SVR patients had post-treatment Child–Pugh score available. Five patients improved from Child B to Child A (B7 to A6 [n = 2] and A5 [n = 3]), and the other 2 patients remained with Child B (B7 to B7 [n = 1] and B8 [n = 1]).

### Subgroup analysis for SVR12

For the SVR rate in selected subgroups of special interest, it was 97.9% (595/608) for patients with baseline HCV RNA level > 6,000.000 IU/mL, 96.1% (148/154) for HIV-coinfection patients, 98.8% (245/248) for HBV-coinfection patients, 90.2% (37/41) for people who inject drugs (PWID), and 100% (6/6) for patients with adherence < 80% (Fig. 3b). The treatment responses in patients with different HCV genotypes stratified by treatment duration are shown in Supplementary Table 1.

Univariate and subsequent multivariate logistic regression analyses showed that age, GLE/PIB treatment duration, treatment adherence, prior antiviral treatment failure, liver cirrhosis, or comorbidities such as HBV or HIV co-infection, HCC, or CKD were not associated with GLE/PIB treatment outcomes. It is also noted that albeit the male gender (odds ratio [OR]/95% confidence intervals [CI]: 3.25/1.31–8.07, *P* = 0.01), baseline HCV RNA levels (OR/CI 1.88/1.19–2.99, *P* < 0.01), HCV GT3 (OR/CI 2.96/1.20–7.29, *P* = 0.02) and intravenous drug abuse (OR/CI 5.51/1.44–21.0, *P* = 0.01) were associated with a low rate of SVR12, the SVR12 rates for all patient subgroups were similarly high (> 95%) with the sole exception for PWID (Table 2).

**Table 2:** Univariate and multivariate logistic regression analysis for predicting factors associated with SVR12.

Predictor	SVR12, n/N (%)	Unadjusted OR (95% CI)	*P* value	Adjusted OR (95% CI)	*P* value
Age		1.05 (1.02–1.07)	< 0.01	1.03 (1.00–1.06)	0.08
**Gender**					
Female	1543/1549 (99.6)	Ref		Ref	
Male	1567/1595 (98.2)	0.22 (0.09–0.53)	< 0.01	0.31 (0.12–0.76)	0.01
**Prior HCV treatment**					
No	2859/2891 (98.9)	Ref			
Yes	251/253 (99.2)	1.40 (0.33–5.90)	0.64		
**HBV coinfection**					
No	2865/2896 (98.9)	Ref			
Yes	245/248 (98.8)	0.88 (0.27–2.91)	0.84		
**HIV coinfection**					
No	2962/2990 (99.1)	Ref		Ref	
Yes	148/154 (96.1)	0.23 (0.10–0.58)	< 0.01	0.95 (0.31–2.94)	0.94
**History of HCC**					
No	2995/3027 (98.9)	Ref			
Active HCC	88/90 (97.8)	0.47 (0.11–1.99)	0.31		
Inactive HCC	27/27 (100.0)	–	–		
**Liver cirrhosis**					
No	2646/2675 (98.9)	Ref			
Yes	464 /469 (98.9)	1.02 (0.39–2.64)	0.97		
**HCV RNA, log**		0.48 (0.30–0.77)	< 0.01	0.53 (0.33–0.84)	< 0.01
≤ 6,000,000 IU/ml	2515/2536 (99.2)	Ref			
> 6,000,000 IU/ml	595/608 (97.9)	0.38 (0.19–0.77)	< 0.01		
**HCV genotype**					
Non-GT3	2971/2998 (99.1)	Ref		Ref	
GT3	139/146 (95.2)	0.18 (0.08–0.42)	< 0.01	0.34 (0.14–0.83)	0.02
**PWID**					
No	3073/3103 (99.0)	Ref		Ref	
Yes	37/41 (90.2)	0.09 (0.03–0.27)	< 0.01	0.18 (0.05–0.69)	0.01
**CKD**					
No	2284/2314 (98.7)	Ref			
Yes	826/830 (99.5)	2.71 (0.95–7.72)	0.06		
**Treatment duration**					
8 week	2570/2601 (98.8)	Ref			
12 week	519/522 (99.4)	2.09 (0.64–6.85)	0.23		
16 week	21/21 (100.0)	–	–		
**Adherence**					
> 80%	3104/3138 (98.9)	–	–		
< 80%	6/6 (100.0)	–	–		

HBV, hepatitis B virus; HIV, human immunodeficiency virus; HCC, hepatocellular carcinoma; CKD, chronic kidney disease; PWID, patients who inject drugs; GT, genotype.

### Safety

As shown in Table 3, 864 patients (27.5%) experienced AEs. The most common AEs (≥ 1% of total patients) were fatigue (7.5%), pruritus (6.7%) and dizziness (1.5%). Eight patients (0.3%) had documented serious AEs (eye ptosis, arrhythmia, upper gastrointestinal tract bleeding, acute on chronic kidney disease, hepatic nodule, HCC, dizziness, recurrent HCC, and colon cancer). The proportion of abnormal liver function were also displayed. The causality of the adverse events and laboratory abnormalities were not presented as its assessment was not mandatory according to the TACR database.

**Table 3:** Patients with adverse events (AEs).

Event, n (%)	All patients (n = 3144)	8-week (n = 2601)	12-week (n = 522)	16-week (n = 21)
Any adverse event	864 (27.5)	636 (24.5)	225 (43.1)	3 (14.3)
Serious adverse event	8 (0.3)	6 (0.2)^a^	2 (0.4)^b^	0 (0.0)
**Adverse event occurring in ≥ 1% of total patients**				
Fatigue	237 (7.5)	181 (7.0)	56 (10.7)	0 (0.0)
Pruritus	210 (6.7)	156 (6.0)	53 (10.2)	1 (4.6)
Dizziness	48 (1.5)	38 (1.5)	10 (1.9)	0 (0.0)
**Total blood bilirubin increased**^**1**^				
Grade 1	15 (0.5)	9 (0.4)	6 (1.2)	0 (0.0)
Grade 2	74 (2.4)	58 (2.2)	16 (3.1)	0 (0.0)
Grade3	9 (0.3)	5 (0.2)	4 (0.8)	0 (0.0)
**Alanine aminotransferase increased**^**2**^				
Grade 1	23 (0.7)	16 (0.6)	7 (1.3)	0 (0.0)
Grade 2	26 (0.8)	19 (0.7)	7 (1.3)	0 (0.0)
Grade 3–4	20 (0.6)	15 (0.6)	5 (0.9)	0 (0.0)
**Aspartate aminotransferase increased**^**2**^				
Grade 1	31 (1.0)	26 (1.0)	4 (0.8)	1 (4.8)
Grade 2	9 (0.3)	6 (0.2)	3 (0.6)	0 (0.0)
Grade 3–4	21 (0.7)	16 (0.6)	5 (1.0)	0 (0.0)

^a^1 patient Rt eye ptosis; 1 patient Arrhythmia; 1 patient Upper gastrointesinal tract bleeding, acute on Chronic kidney disease;1 patient Liver nodule;1 patient Hepatocellular carcinoma;1patient dizziness.

^b^1 patient Hepatocellular carcinoma recurrent; 1 patient Colon cancer.

Adverse Events graded based on CTCAE 5.0.

^1^Total Blood bilirubin increased: Grade1: 1.0 ULN -1.5 × ULN if baseline was normal; > 1.0 to 1.5 × baseline if baseline was abnormal; Grade2: > 1.5 to 3.0 × ULN if baseline was normal; > 1.5 to 3.0 × baseline if baseline was abnormal; Grade3: > 3.0 − 10.0 × ULN if baseline was normal; > 3.0 − 10.0 × baseline if baseline was abnormal.

^2^Alanine aminotransferase/ Aspartate aminotransferase increased: Grade1: > 1.0 to 3.0 × ULN if baseline was normal; > 1.5 to 3.0 × baseline if baseline was abnormal; Grade2: > 3.0 − 5.0 × ULN if baseline was normal; > 3.0–5.0 × baseline if baseline was abnormalGrade3: > 5.0 − 20.0 × ULN if baseline was normal; > 5.0 − 20.0 × baseline if baseline was abnormal; Grade4: > 20.0 × ULN if baseline was normal; > 20.0 × baseline if baseline was abnormal.

### Health care resource utilization

The mean number (mean ± SD) of outpatient visits during the GLE/PIB treatment (from GLE/PIB initiation to SVR 12 survey visit) was 6.11 ± 1.00 visits for all patients, with 5.94 ± 0.88 visits for patients receiving 8-week regimen, 6.90 ± 1.16 visits for patients receiving 12-week regimen, and 6.77 ± 1.07 visits for patients receiving 16-week regimen. There was a statistically significant difference in the number of visits between the patients receiving 8-week and 12-week regimens (*P* < 0.0001).

## Discussion

Real-world data and the results from clinical studies are complementary to each other, both provide valuable information in routine clinical practice. The result of this nation-wide, large-scaled study indicates that GLE/PIB is an effective and well-tolerated pangenotypic DAA for Taiwanese patients with CHC infection irrespective of host or viral diversities in the real-world setting.

The characteristics of this patient cohort were generally representative, as the majority of the 3144 patients were non-cirrhotic (85.1%) and had not undergone other HCV treatment prior to GLE/PIB (92.0%). In accordance with the TFDA-approved label at the time of enrollment, 2601 (82.7%) of the patients received 8-week treatment regimen. Similar to the previous real-world reports of GLE/PIB, a large part (56.8%) of the current cohort had GT2 infection, in contrast to the overall genotype distribution in Taiwan, which was dominant by GT1^13,14^. The preponderance of GT2 CHC in GLE/PIB reports reflected the evolution of DAAs, that the treatments of GT1 CHC were licensed earlier than the treatments of other genotypes^14^.

The overall SVR12 rate of 98.9% was comparable with the registrational phase II and III clinical studies^10,11^ and previously published real-world reports^13–15,17^. Even when stratified according to cirrhosis status and treatment experience, GLE/PIB demonstrated a similarly high SVR12 rate of 98.5% in the more difficult-to-treat patients who were cirrhotic and had exposed to previous HCV treatment.

In addition, this study reinforced the effectiveness of GLE/PIB in several subpopulations of interest, including patients with HIV or HBV dual infections or with comorbidities such as HCC or CKD. The favorable treatment outcome in CHC patients dually infected with HIV has been previously proven in clinical trials and real-world reports^10,13^. Unlikely most western countries, both HBV and HCV are endemic in Taiwan^18^. However, the treatment efficacy of GLE/PIB in patients with HBV/HCV dual infection has rarely been validated on a large population basis^13,19^. In the present cohort, the SVR12 rate of the 248 patients (7.9%) with HBV/HCV dual infection was equally high (98.8%) as in the HCV mono-infected patients (98.9%).

It has been previously reported that patients with active HCC are prone to encounter DAA treatment failure^15,20^, yet the information is scarce with patients who received GLE/PIB. Ninety patients (2.9%) of the present cohort had documented active HCC, and the SVR12 rate of 97.8% was comparable to the patients without a history of HCC (98.9%) or with inactive HCC (100%). As for the 830 patients (26.4%) with renal impairment, the high SVR12 rate of 99.5% was also in line with the previous observations of GLE/PIB^13,17,21^. The results of the univariate and subsequent multivariate logistic regression analyses in this study also indicated that the comorbidities of HCC or CKD were not associated with the efficacy outcome of GLE/PIB.

It is important to report the potential factors associated with a lower SVR12 rate. In contrast to the data reported in registration trials^10^, lower SVR rates were observed to be associated with males, high baseline HCV RNA levels, HCV GT3 and intravenous drug abuse in the current study. Other large-scaled real-world reports and post marketing observational studies for GLE/PIB have reported conflicting results of the predicting factors including the male gender^22,23^, HCV viral load at baseline^23,24^, GT3^24,25^ and people who use drugs^22,25^. It is worth noting that the SVR12 rates remained high (> 95%) for all subgroups analyzed, excepting a numerically lower SVR12 rate of 90.2% for PWID, but it might have been affected by the small number of patients (41, 1.3%).

The phase 3 EXPEDITION-8 trial reported that for the treatment-naïve patients with compensated cirrhosis, 8 weeks of GLE/PIB achieved a high SVR12 rate of 99.7%^8^. Based on this trial, an 8-week GLE/PIB regimen for treatment of cirrhotic patients who received no prior HCV treatment was approved by TFDA in April 2020. It is hoped that reducing treatment duration may help to address remaining gaps in the cascade of care of HCV^4^, yet there have only been very limited real-world studies of the effect of the 8-week GLE/PIB regimen on the treatment-naïve patients with compensated cirrhosis^23,26,27^. The present study demonstrated that 8 weeks of GLE/PIB achieved a high SVR12 rate (98.2%) in 110 treatment-naïve Taiwanese patients with compensated cirrhosis, supporting the results of EXPEDITION-8^8^.

No specific safety issues were observed from GLE/PIB initiation to the SVR12 survey visit, and the common adverse events were similar to what had been reported in clinical studies^11^ or real-world publications^17,27^.

Compared with the 12-week regimen, 8-week GLE/PIB was associated with a reduction in healthcare resource utilization (5.94 visits vs. 6.90 visits), determined by the number of clinic visits. In consistence with previous reports, the shorter, 8-week treatment with GLE/PIB can reduce healthcare resource use, which may further reduce the health and cost burden of the disease^28^.

Real-world observational studies such as this have inherent limitations. Firstly, the treatment outcomes for certain populations may be inconclusive due to limited patient numbers, such as patients who injected drugs, with HCV GT 4 or 5 infection, or receiving 16-week regimen. Secondly, for the treatment naïve patients with compensated cirrhosis, the 8-week treatment emerged only very recently, and thus only a small portion of such patients were treated for the shorter course. Lastly, the information of drug abuse and data of adverse events were subject to reporting biases, and the causal relationships between the AEs and the treatment could not be fully established. The information regarding the issue of HBV reactivation among HBV/HCV dually infected patients was also not available in the national registry.

In conclusion, the result of this study demonstrated that based on the first and largest real-world, nation-wide registry in Taiwan, GLE/PIB was highly effective and safe in treating CHC patients across viral genotypes and special subgroups including treatment-experienced or cirrhotic patients. This study also adds to the growing body of evidence supporting that the shorter, 8-week GLE/PIB regimen may be an effective and cost-saving pangenotypic treatment option for the majority of patients with CHC infection.

## Supplementary Information

Supplementary Information.

## Abbreviations

CHC: Chronic hepatitis C
DAA: Direct-acting antiviral agents
GLE: Glecaprevir
HCC: Hepatocellular carcinoma
HCV: Hepatitis C virus
PIB: Pibrentasvir
SAE: Serious adverse event
SVR: Sustained virological response
