# Supplementary material for: Nationwide registry of glecaprevir plus pibrentasvir in the treatment of HCV in Taiwan
Source: Sci Rep. 2021 Dec 6;11:23473. doi: 10.1038/s41598-021-03006-3 (PMC8648748; doi:10.1038/s41598-021-03006-3)
Supplement: Supplementary file 1 — Supplementary Information. [file 41598_2021_3006_MOESM1_ESM.pdf]

Supplementary Table 1. SVR stratified by different treatment durations

Characteristics	All patients (n=3,144)			8-week (n=2,601)			12-week (n=522)			16-week (n=21)		
	**Patient No.**	**SVR12 (%)**	**95% CI**	**Patient No.**	**SVR12 (%)**	**95% CI**	**Patient No.**	**SVR12 (%)**	**95% CI**	**Patient No.**	**SVR12 (%)**	**95% CI**
Overall	3,144	3,110 (98.9)	98.5-99.2	2,601	2,570 (98.8)	98.3-99.2	522	519 (99.4)	98.3-99.8	21	21 (100.0)	84.5-100.0
Genotype												
GT3	146	139 (95.2)	90.4-97.7	105	100 (95.2)	89.3-97.9	31	29 (93.5)	79.3-98.2	10	10 (100.0)	72.2-100.0
Non-GT3	2,998	2,971 (99.1)	98.7-99.4	2,496	2,470 (99.0)	98.5-99.3	491	490 (99.8)	98.9-99.9	11	11 (100.0)	74.1-100.0
GT1	864	859 (99.4)	98.7-99.8	741	736 (99.3)	98.4-99.7	112	112 (100.0)	96.7-100.0	11	11 (100.0)	74.1-100.0
GT2	1,785	1,767 (99.0)	98.4-99.4	1,456	1,438 (98.8)	98.1-99.2	329	329 (100.0)	98.8-100.0	0	0 (0.0)	-
GT4	1	1 (100.0)	20.7-100.0	0	0 (0.0)	-	1	1 (100.0)	20.7-100.0	0	0 (0.0)	-
GT5	1	1 (100.0)	20.7-100.0	1	1 (100.0)	20.7-100.0	0	0 (0.0)	-	0	0 (0.0)	-
GT6	266	262 (98.5)	96.2-99.4	239	236 (98.7)	96.4-99.6	27	26 (96.3)	81.7-99.3	0	0 (0.0)	-
Mixed	51	51 (100.0)	93.0-100.0	35	35 (100.0)	90.1-100.0	16	16 (100.0)	80.6-100.0	0	0 (0.0)	-
Unclassified	30	30 (100.0)	88.6-100.0	24	24 (100.0)	86.2-100.0	6	6 (100.0)	61.0-100.0	0	0 (0.0)	-
Adherence												
80-100%	3,138	3,104 (98.9)	98.5-99.2	2,596	2,565 (98.9)	98.3-99.2	521	518 (99.4)	98.3-99.8	21	21 (100.0)	84.5-100.0
<80%	6	6 (100.0)	61.0-100.0	5	5 (100.0)	56.6-100.0	1	1 (100.0)	20.7-100.0	0	0 (0.0)	-
HIV coinfection												
No	2,990	2,962 (99.1)	98.6-99.4	2,453	2,428 (99.0)	98.4-99.3	517	514 (99.4)	98.3-99.8	20	20 (100.0)	83.9-100.0

Yes	154	148 (96.1)	91.8-98.2	148	142 (95.9)	91.4-98.1	5	5 (100.0)	56.6-100.0	1	1 (100.0)	20.7-100.0
HBV coinfection												
No	2,896	2,865 (98.9)	98.5-99.2	2,406	2,378 (98.8)	98.3-99.2	470	467 (99.4)	98.1-99.8	20	20 (100.0)	83.9-100.0
Yes	248	245 (98.8)	96.5-99.6	195	192 (98.5)	95.6-99.5	52	52 (100.0)	93.1-100.0	1	1 (100.0)	20.7-100.0
PWID												
No	3,103	3,073 (99.0)	98.6-99.3	2,563	2,536 (98.9)	98.5-99.3	519	516 (99.4)	98.3-99.8	21	21 (100.0)	84.5-100.0
Yes	41	37 (90.2)	77.5-96.1	38	34 (89.5)	75.9-95.8	3	3 (100.0)	43.9-100.0	0	0 (0.0)	-
Prior antiviral retreatment												
Naive	2,891	2,859 (98.9)	98.4-99.2	2,428	2,399 (98.8)	98.3-99.2	458	455 (99.3)	98.1-99.8	5	5 (100.0)	56.6-100.0
Experienced	253	251 (99.2)	97.2-99.8	173	171 (98.8)	95.9-99.7	64	64 (100.0)	94.3-100.0	16	16 (100.0)	80.6-100.0
Liver Cirrhosis												
Non-cirrhosis	2,675	2,646 (98.9)	98.4-99.2	2,478	2,450 (98.9)	98.4-99.2	188	187 (99.5)	97.0-99.9	9	9 (100.0)	70.1-100.0
Cirrhosis	469	464 (98.9)	97.5-99.5	123	120 (97.6)	93.1-99.2	334	332 (99.4)	97.8-99.8	12	12 (100.0)	75.8-100.0
Prior HCV Treatment x Liver Cirrhosis												
Naïve x non-cirrhosis	2,487	2,459 (98.9)	98.4-99.2	2,318	2,291 (98.8)	98.3-99.2	167	166 (99.4)	96.7-99.9	2	2 (100.0)	34.2-100.0
Naïve x cirrhosis	404	400 (99.0)	97.5-99.6	110	108 (98.2)	93.3-99.5	291	289 (99.3)	97.5-99.8	3	3 (100.0)	43.9-100.0
Experience x non-cirrhosis	188	187 (99.5)	97.0-99.9	160	159 (99.4)	96.5-99.9	21	21 (100.0)	84.5-100.0	7	7 (100.0)	64.6-100.0
Experience x cirrhosis	65	64 (98.5)	91.8-99.7	13	12 (92.3)	66.7-98.6	43	43 (100.0)	91.8-100.0	9	9 (100.0)	70.1-100.0

Note: HBV, hepatitis B virus; HIV, human immunodeficiency virus; HCC, hepatocellular carcinoma; CKD, chronic kidney disease; PWID, patients who inject drugs; GT, genotype.
